# Selective effect of phosphatidylcholine on the lysis of adipocytes

**DOI:** 10.1371/journal.pone.0176722

**Published:** 2017-05-02

**Authors:** Ji-Young Kim, Min-Seo Kwon, Junghyun Son, Sang-Wook Kang, Youngsup Song

**Affiliations:** 1 Department of Biomedical Sciences, University of Ulsan College of Medicine, Asan Institute for Life Sciences, Asan Medical Center, Seoul, Korea; 2 Bio-Medical Institute of Technology, University of Ulsan, College of Medicine, Seoul, Korea; 3 Department of Biological Chemistry, Korea University of Science and Technology, Daejeon, Korea; 4 Doping Control Center, Korea Institute of Science and Technology, Seoul, Korea; University of Cambridge, UNITED KINGDOM

## Abstract

Obesity, a serious health risk factor, is often associated with depression and negatively affects many aspects of life. Injection of a formula comprising phosphatidylcholine (PPC) and deoxycholate (DC) has emerged as an alternative to liposuction in the reduction of local fat deposits. However, the formula component mainly responsible for this effect and the mechanism behind the actions of the components with respect to fat reduction are unknown. Here, we investigate the specific effects of PPC and DC on adipocyte viability. When exposed to PPC or DC, 3T3L1 preadipocytes and differentiated adipocytes showed dose dependent decrease in cell viability. Interestingly, while DC mediated cell death was non-specific to both preadipocytes and adipocytes, PPC specifically induced a decrease in mature adipocyte viability, but had less effect on preadipocytes. Injection of PPC and DC into inguinal fat pads caused reduction in size. PPC injections preferentially decreased gene expression in mature adipocytes, while a strong inflammatory response was elicited by DC injection. In line with the decreased adipocyte viability, exposure of differentiated adipocytes to PPC resulted in triglyceride release, with a minimal effect on free fatty acids release, suggesting that its fat-reducing effect mediated mainly through the induction of adipocyte cell death rather than lipolysis. Taken together, it appears that PPC specifically affects adipocytes, and has less effect on preadipocyte viability. It can therefore be a promising agent to selectively reduce adipose tissue mass.

## Introduction

More than 30% of the US population suffers from obesity. It is a serious risk factor that can induce or exacerbate many metabolic disorders, including cardiovascular disease, dyslipidemia, and diabetes. It has also been associated with mental health problems; there is an increased prevalence of depression among obese patients [1], which in turn negatively affects many aspects of life and can result in impaired social behavior, eating disorders, and lower self-esteem [2]. Therefore, the reduction of adipose tissue is important for solving medical issues as well as for aesthetic purposes.

Surgical methods for removing adipose tissue, such as liposuction, have been developed over the years. Although liposuction does not seem to produce long-term improvements in weight loss or metabolic parameters, it has become one of the most effective procedures with respect to the removal of localized fat deposits for aesthetic purposes [3]. However, as with any surgery, liposuction requires anesthesia and poses a risk of medical complications, even leading to death on rare occasions [4]. Injection of Lipostabil (Nattermann-Aventis Pharma, Germany), a drug originally introduced to prevent or attenuate hepatic steatosis, has emerged as a non-surgical alternative to liposuction. In fact, several clinical studies have reported that Lipostabil is highly efficient in reducing localized fat deposits by stimulating lysis or lipolysis in adipose tissue [5–7]. Lipostabil comprises two main components, phosphatidylcholine (PPC) and deoxycholate (DC). PPC is a lecithin-derived phospholipid that can be abundantly found in egg yolk, milk, sunflower seeds, and soybeans. Structurally, it has two fatty acids attached to the first two carbons and phosphoric choline attached to the third carbon; it is widely used as an emulsifier in food manufacturing. Due to its lipid-lowering effect in liver and serum, PPC was thought to be the functional component responsible for the lysis of fat [8, 9], while DC, a bile salt, was thought to be an auxiliary component, which forms micelles and facilitates aqueous solubility of PPC [10, 11]. However, there are also reports suggesting that DC, and not PPC, is the major active ingredient responsible for the reduction of adipose tissue [12, 13]. These studies, which compared the effects of a combined PPC/DC formula with those of DC alone, found that PPC/DC and DC induced a comparable level of fat reduction and that DC was responsible for fat-reducing effects of the combination formula. However, since they did not compare the individual effects of PPC to DC, the fat-reduction ability of PPC is still unknown. Furthermore, DC is a widely used laboratory detergent and the use of pure DC can damage adipose tissue other than the targeted adipocytes. It can also induce adverse effects including inflammatory responses and injuries to the nervous system.

In this study, we utilized both *in vitro* methods and an *in vivo* rat model to compare the fat-reducing activities of PPC and DC, and tested their adipocyte specificity.

## Materials and methods

### Reagents

**Fig 1:**
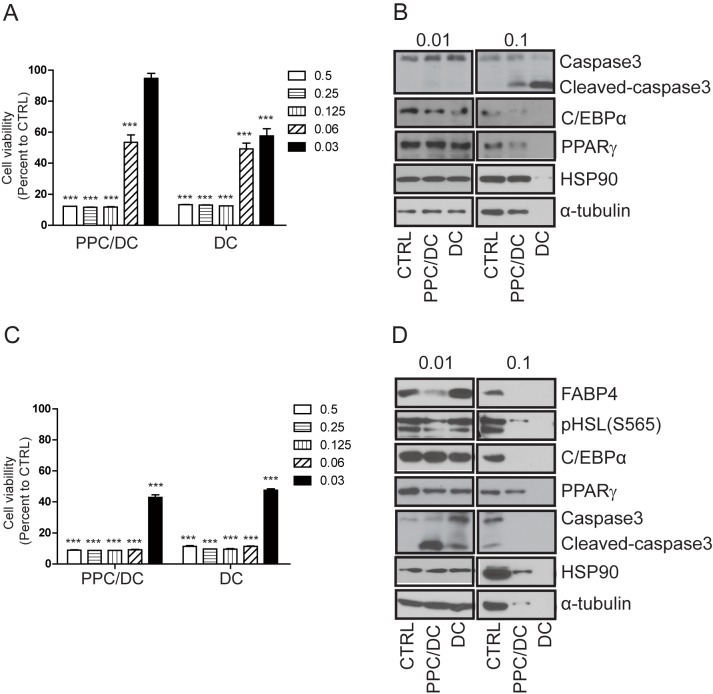
Effects of the phosphatidylcholine (PPC)/deoxycholate (DC) formula or DC alone on the viability of preadipocytes and adipocytes. A. 3T3L1 preadipocyte and B. adipocyte cell viability was measured eight hours after treatment with various doses of the PPC/DC formula or DC only using the MTT assay. Protein samples from C. 3T3L1 preadipocytes and D. adipocytes following 0.01% or 0.1% PPC/DC formula or DC treatment for eight hours, respectively, were prepared and analyzed by Western blotting.

PPC (97.8% pure polyene phosphatidylcholine) and sodium DC (99.1% pure) were acquired from Lipoid (Lipoid Kosmetik AG, Switzerland), and NZP (New Zealand Pharmaceuticals, Ltd., New Zealand), respectively. The PPC/DC formula shown in Fig 1 contains 5% PPC and 2.4% sodium DC in water, and the DC solution shown in the Figure contains 2.4% sodium DC. For PPC and DC treatment in 3T3L1 preadipocytes, adipocytes, and rats, 5% PPC and DC stock solutions were prepared in ethanol and water, respectively.

### Animals and experimental design

All animal studies were conducted according to a protocol approved by the Institutional Animal Care and Use Committee (IACUC) of the Asan Life Science Institute, Asan Medical Center, Seoul, Korea. Sprague Dawley rats (8–10 weeks old), weighing around 300 g, were purchased from Orientbio (Seongnam, Republic of Korea), and raised under temperature-controlled specific-pathogen-free (SPF) conditions with a 12-h light/12-h dark cycle (lights switched on at 8:00 AM and switched off at 8:00 PM) and free access to water and a normal chow diet (Purina Rodent Chow, 38057). Rats aged between 10 and 12 weeks, were randomly divided into four groups, and the bilateral inguinal fat pad area was assessed using magnetic resonance imaging (MRI) before PPC or DC injection and seven, fourteen, and twenty eight days after injection. For PPC or DC injection, a 2-cm incision was made at the bilateral inguinal fat pad area. The left side of the fat pad was treated with either 25 mg PPC, 12.5 mg PPC, 25 mg DC, or 12.5 mg DC by uniformly dividing the treatment into four or five injections. The right side was treated with the same volumes of ethanol (for PPC) or phosphate-buffered saline (PBS; for DC) as a control. After injection, the incised region was sutured and the area injected with PPC or DC (on the left side) and that treated as a control (on the right side) were compared by MRI analysis.

### MRI analysis

Approximately 10 images were taken to cover the entire inguinal fat pad area for each animal, before injection and 7, 14, and 28 days after injection. After collecting all images, a blind analysis for measuring the inguinal fat area was carried out using imageJ with the fat intensity (white intensity) threshold set at 10,000.

### 3T3L1 culture and adipocyte differentiation

The 3T3L1 cell line was cultured in Dulbecco's Minimal Essential Medium supplemented with 10% fetal bovine serum (HyClone, Logan, UT, USA) and 1% penicillin-streptomycin. For adipocyte differentiation, 3 ×10^5^ 3T3L1 cells were plated in 12-well plates. Two days after plating, adipogenesis was induced by switching to a 3T3L1 culture medium supplemented with 4 mg/ml dexamethasone, 0.5 μM IBX, 0.5 unit/ml Humulin (Lilly, USA), and 100 nM rosiglitazone (Roche, Switzerland). Two days after exposure of the cells to the adipogenic induction medium, this was replaced with fresh 3T3L1 culture medium containing only 0.5 unit/ml Humulin and 100 nM rosiglitazone. Fresh medium was added every two days for further 5 or 6 days. Adipogenesis was determined by light microscopy assessment of lipid vacuole formation and Oil Red O staining.

### Cell viability assay

PPC or DC stock (5%) solutions were diluted to make 1000 × for each concentration and were applied to the cell culture medium, so that the final concentration of the solvent (ethanol for PPC and water for DC) exposed to the cells was 0.1%. Cell viability was assessed using the MTT assay. Briefly, the day before the experiment, 10 × 10^4^ cells were plated in 24-well tissue culture plates. On the day of the experiment, PPC, DC or the control (ethanol as the PPC control and water as the DC control) was added as indicated in the figure legends; one hour before the termination of the experiment, 5 mg/ml of MTT (Duchefa Biochemie, Netherlands) was applied. Cells were washed with PBS, and 150 μl DMSO was applied for 15 min to extract the dye. Cell viability was estimated by measuring absorbance at 565 nm using a plate reader (BioTek, USA).

### Western blotting and antibodies

The day prior to experimental day, 3 ×10^5^ 3T3L1 preadipocyte cell were plated in 12-well plates, and the PPC/DC formula, PPC, or DC was applied as indicated in the figures. The 3T3L1 preadipocyte were differentiated to adipocytes in accordance with the adipocyte differentiation protocol outlined above. After confirmation of adipocyte differentiation by visualization of lipid vacuole formation using Oil Red O staining, differentiated adipocytes were treated with the PPC/DC formula, PPC, or DC. Protein samples were prepared in 120 μl of lysis buffer (10 mM Tris (pH 7.4), EDTA, and 1% SDS supplemented with a proteinase inhibitor (Roche)) per well of a 24 well plate. For immunoblot analysis, primary antibodies for PPARγ, C/EBPα, FABP4, α-tubulin, HSP90 α/β (Santa Cruz Biotechnology, Inc., Santa Cruz, CA, USA), PARP, caspase-3, and phospho-hormone sensitive lipase (HSL) (Cell Signaling Technology, Danvers, MA, USA) and secondary antibodies for HRP-conjugated anti-rabbit or anti-mouse IgG (Thermo Fisher, USA) were used.

### Histology

Isolated tissue from rats was immediately fixed with 4% paraformaldehyde and embedded in paraffin. Sections (5-μm thick) were stained with hematoxylin and eosin or immunohistochemical staining was carried out using the F4/80 antibody (Abcam, Cambridge, UK) followed by the application of the avidin-biotin, DAB staining method (Vector Laboratories, Burlingame, CA, USA).

### mRNA expression analysis

Total RNA was prepared and the first cDNA synthesized was used for mRNA expression as described previously [14]. Briefly, immediately after dissection, rat tissue was frozen in liquid nitrogen and kept at -80°C for further processing. The tissue samples were ground in liquid nitrogen and total RNA was extracted using the Tri-RNA reagent according to the manufacturer's instructions. About 500 ng of total RNA was used for the first cDNA synthesis (Toyobo, Osaka, Japan), and mRNA expression was quantified using quantitative reverse transcription-PCR (qRT-PCR) with SYBR Green PCR mix (Toyobo, Osaka, Japan). The following primers were used for the specific amplification and quantification of gene expression with real-time RT-PCR.: EMR1; EMR1-F: 5′-AGGGCCTGGAAGAATCTTGT-3′, and EMR1-R: 5′-CGTGTTGATGCAAATGAAGG-3′, IL1β; IL1β-F: 5′-AAATACCTGTGGCCTTGGGC-3′, and IL1β-R: 5′- CTTGGGATCCACACT CTCCAG-3′, and FABP4; FABP4-F: 5′-TCTGGTGAAGCC CAAGATCG-3′, and FABP4-R: 5′-CCTCTGGGTTTCCGCCAGTT-3′.

### Statistics

Data are presented as the mean ± standard error of the mean (SEM), and statistical analysis was carried out using an unpaired Student’s t-test and GraphPad Prism. In this study, *p*<0.05, *p*<0.01, and *p*<0.001 indicated by *, **, and *** respectively, were considered statistically significant.

## Results

### Effects of PPC/DC formula and DC on lysis of adipocytes

To examine whether the fat-reducing effect of Lipostabil (the PPC/DC formula) was mediated by PPC or DC, we compared cell viability after treatment with the PPC/DC formula (5% PPC and 2.4% DC) or with 2.4% DC only. Both the PPC/DC formula and the DC-only treatments decreased cell viability in 3T3L1 preadipocyte cells in a dose-dependent manner. However, the DC-only treatment appeared to exert a stronger effect than the PPC/DC formula treatment, since compared with the formula, which at a 0.03% concentration decreased 3T3L1 preadipocyte viability by only 6%, 0.03% of the DC-only treatment decreased cell viability by 43% (Fig 1A). Consistent with these results, an eight-hour treatment with 0.1% PPC/DC formula or 0.1% DC decreased protein levels in 3T3L1 preadipocytes as measured by Western blot analysis; again, a greater decrease in protein levels was observed in samples treated with DC only. The downregulation of protein levels appeared to be caused by cell death, as both treatments decreased these levels non-specifically including the levels of proteins related to housekeeping genes, such as HSP90 and α-tubulin (Fig 1B). We then compared adipocyte viability after treatment with the PPC/DC formula or DC only. For this experiment, we differentiated the 3T3L1 preadipocytes into adipocytes, treated them with the PPC/DC formula or DC only, and measured cell viability. The results of the MTT assay indicated that the PPC/DC formula and the DC-only treatments caused a comparable decrease in adipocyte viability (Fig 1C). Interestingly, we observed that while the 0.03% DC treatment had a similar effect on the viability of either preadipocytes or adipocytes, the PPC/DC formula had a stronger effect on adipocytes than on preadipocytes (Fig 1A and 1C). Degradation of proteins, including the loading controls HSP90 and α-tubulin, was observed in protein samples from adipocytes treated with a 0.1% concentration of the PPC/DC formula or DC, suggesting that both strongly induced adipocyte cell death. However, at lower doses (0.01%), the PPC/DC formula and not DC, decreased the levels of FABP4, a protein specifically expressed in mature adipocytes. These results, taken together with the data from the cell viability assay, suggest that PPC/DC has a stronger and specific tendency to induce adipocyte cell death compared to DC-only treatments.

### PPC has higher specificity toward adipocytes than DC

**Fig 2:**
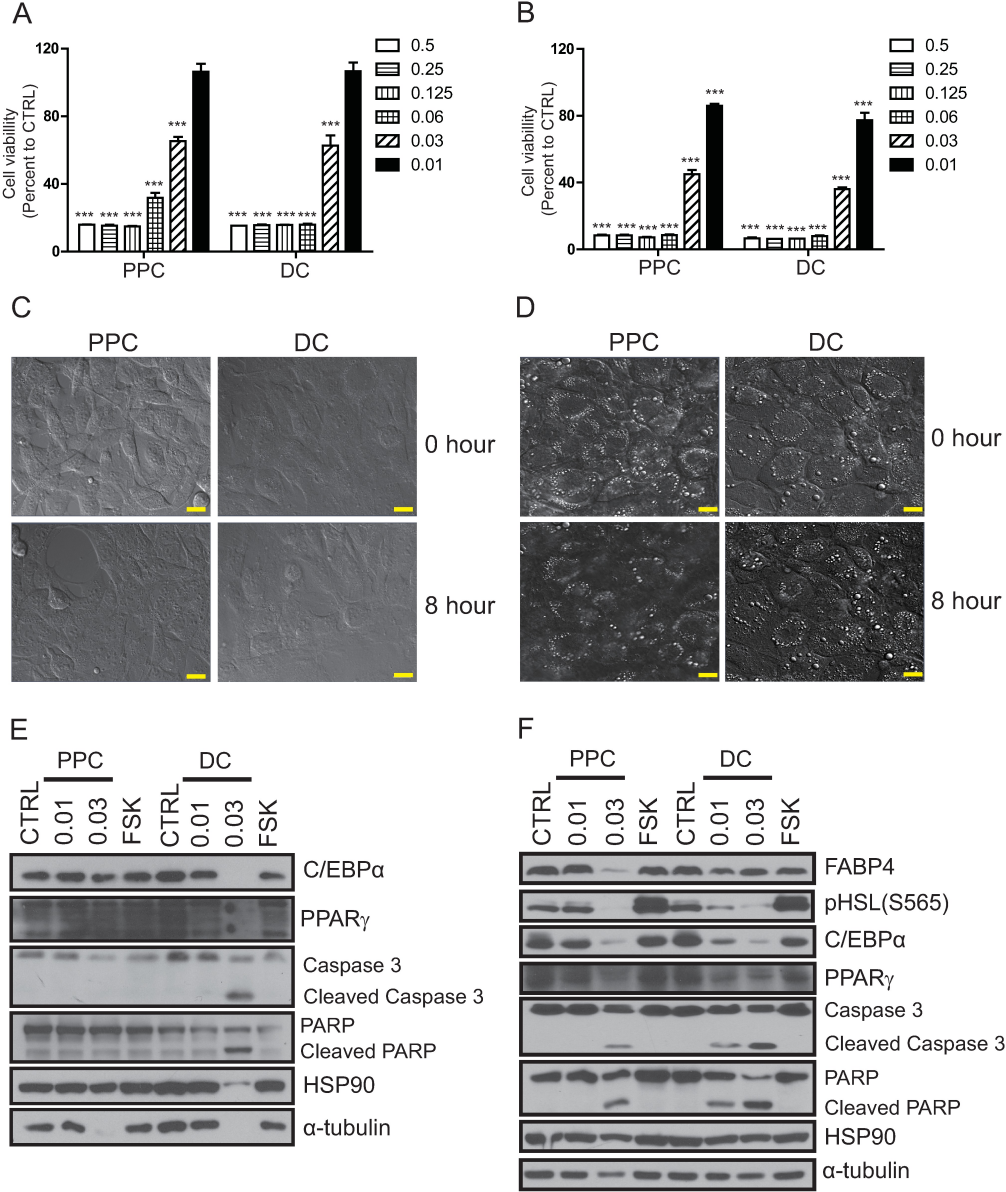
Effect of phosphatidylcholine (PPC) or deoxycholate (DC) on the viability of preadipocytes and adipocytes. A. 3T3L1 preadipocyte and B. adipocyte viability was measured eight hours after treatment with various doses of PPC or DC using the MTT assay. Live confocal images of C. 3T3L1 preadipocytes and D. adipocytes captured before (0 h) and eight hours of treatment with 0.03% of PPC or DC. The white dots indicate the lipid vacuoles of mature adipocytes, and the eight-hour treatment with PPC decreased the number of lipid vacuole-positive cells to a greater extent than DC treatment. Protein samples from E. 3T3L1 preadipocyte and F. adipocytes treated with 0.01% or 0.03% of PPC or DC respectively for eight hours, were prepared and the expression of preadipocyte (C/EBPα, PPARγ, and HSL), and mature adipocyte (C/EBPα, PPARγ, HSL, and FABP4) markers, apoptotic markers (cleaved-caspase3 and cleaved-parp), as well as the amount of loading controls (HSP90 and α-tubulin) were analyzed using Western blotting. The scale bar indicates 20 μm.

As the PPC/DC formula appeared to have a higher efficiency with respect to the lysis of adipocytes than DC alone, we investigated whether PPC was the active component responsible for this. To test this, we performed a cell viability assay comparing the individual effects of PPC and DC. We found that, while the decrease in viability of 3T3L1 preadipocytes when treated with DC was greater than that seen when cells were treated with PPC (Fig 2A), the viability of differentiated adipocytes decreased by a comparable amount when treated with PPC or DC (Fig 2B). We also observed that PPC treatment induced a decrease in lipid vacuoles, which are specifically found in mature adipocytes, as indicated by the reduced number of cells stained positive with Oil Red O (S1 Fig and S2 Fig). Live-cell confocal imaging also showed that during an eight-hour treatment, PPC induced a stronger reduction in lipid vacuoles in adipocytes compared to DC (Fig 2C and 2D, S1–S4 Video files).

Consistent with the cell viability assay and the microscopic observations, both PPC and DC at a concentration of 0.03% induced a non-selective decrease in protein levels, including levels of HSP90 and α-tubulin, in 3T3L1 preadipocytes. This effect was greater following DC treatment (Fig 2E). By contrast, in adipocytes, although both PPC and DC treatments decreased the expression of C/EBPα and PPARγ which are markers for both preadipocytes and mature adipocytes, only PPC treatments decreased FABP4 expression, a marker of mature adipocyte cells (Fig 2F). To test whether the reduction of lipid vacuoles in adipocytes by PPC was mediated through the lipolysis pathway, we examined the phosphorylation (activation) status of HSL after PPC treatment. Administration of forskolin, which stimulates lipolysis through PKA-dependent activation of HSL, induced phosphorylation of HSL without decreasing protein levels in adipocytes; however PPC treatment did not upregulate HSL phosphorylation compared to that seen following control treatment (Fig 2F). In line with this, while the treatment of adipocytes with PPC led to only a 20% increase in free fatty acid release, triglyceride release was increased four-fold compared to that observed in adipocytes treated with the control ethanol solution. These results suggest that PPC decreases the number of lipid vacuoles by inducing adipocyte cell death leading to the release of triglycerides (S3 Fig). As approximately 30–40% of preadipocytes are fully differentiated into mature adipocytes, these data taken together suggest that DC non-specifically induces the death of both 3T3L1 preadipocytes and adipocytes, while PPC acts selectively on adipocytes, and to a lesser extent on preadipocytes (Fig 2E and 2F).

### Reduction of adipose tissue by PPC or DC in a rat model

**Fig 3:**
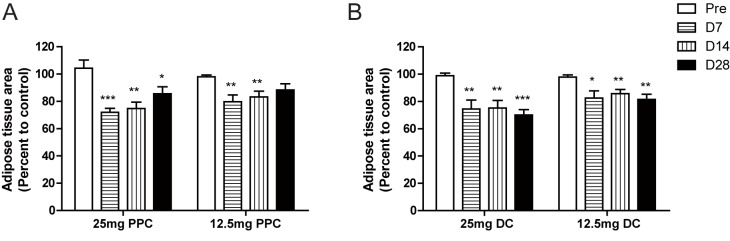
*In vivo* analysis of the inguinal adipose tissue area before and after phosphatidylcholine (PPC) or deoxycholate (DC) injection. MR images were acquired to measure the inguinal fat pad area of Sprague-Dawley rats, before (Pre) and 7, 14, and 28 days after injection of 25 mg of PPC (n = 6), 12.5 mg of PPC (n = 6), 25 mg of DC (n = 5), or 12.5 mg of DC (n = 5) into one side of the bilateral inguinal fat pad and control solution (ethanol for PPC and PBS for DC) into the other side. Data are presented as the percentage of the inguinal fat pad area injected with PPC or DC to the control-injected fat pad area.

**Fig 4:**
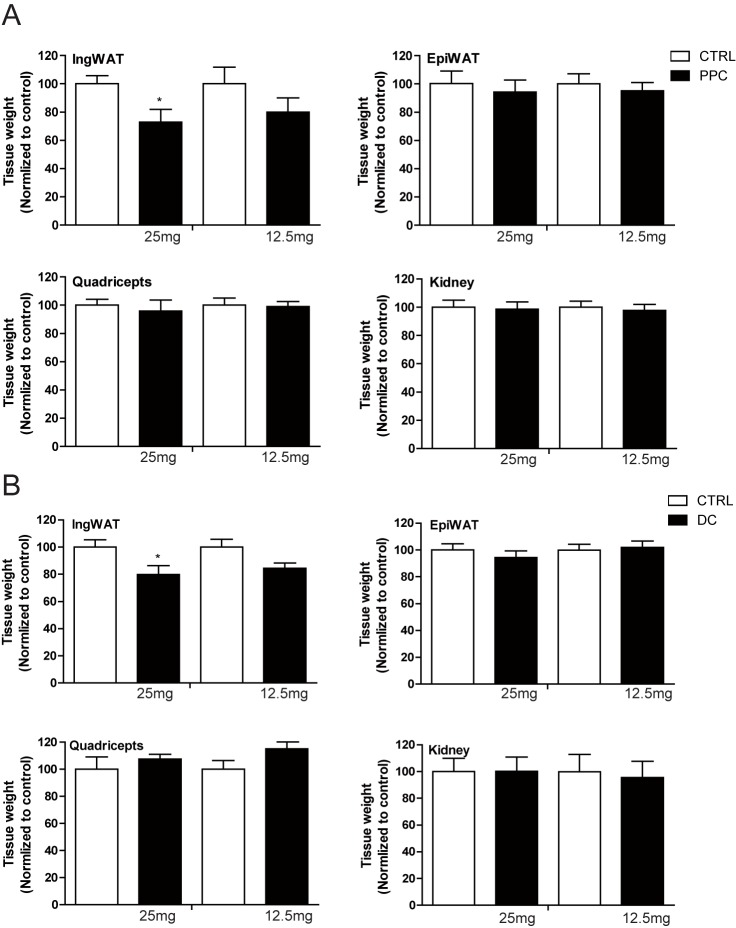
Tissue weights of Sprague-Dawley rats 30 days after phosphatidylcholine (PPC) or deoxycholate (DC) injection. Weights of bilaterally excised tissue, including inguinal adipose tissue (IngWAT), epididymal adipose tissue (EpiWAT), quadriceps muscle, and tissue from the kidneys, were measured 30 days after injection of 25 mg PPC (n = 6), 12.5 mg PPC (n = 6), 25 mg DC (n = 5), or 12.5 mg DC (n = 5). Data are presented as the percent tissue weight resulting from PPC or DC injection to tissue weight resulting from injection of the control solution.

Having observed the effect of PPC on the lysis of adipocytes, we subsequently examined whether PPC or DC could reduce fat deposits in an *in vivo* animal model by locally injecting PPC or DC into the inguinal fat pads of Sprague-Dawley rats. Although we used age-matched rats, there were slight differences in body weight, and therefore, probable differences in body fat as well. To minimize between-animal variation and to analyze the effects of PPC or DC more precisely, we injected one side of the bilateral inguinal fat pad with PPC or DC and the other side with a control solution (ethanol for PPC and PBS for DC). We then compared the reduction in fat caused by PPC or DC with that caused by the control solution in each animal using MRI. Both PPC and DC injections significantly reduced inguinal fat compared to control injections We observed that the decrease in fat caused by PPC peaked at seven days after injection and the fat tissue recovered slightly after four weeks, while fat reduction was maintained for four weeks after DC injection (Fig 3A, 3B and S4 Fig). Thirty days after injection, we euthanized the rats; dissected the tissue, which was present bilaterally, and compared the weight of each tissue. The weight of the inguinal fat pad injected with PPC or DC was decreased compared to the control-injected inguinal fat pad (Fig 4A and 4B). However, the weights of epididymal white adipose tissue, quadriceps muscle, and kidney tissue were comparable, suggesting that the effect of the injection was locally restricted and specific to the injection sites.

### DC injection elicits a stronger inflammatory response than does PPC

**Fig 5:**
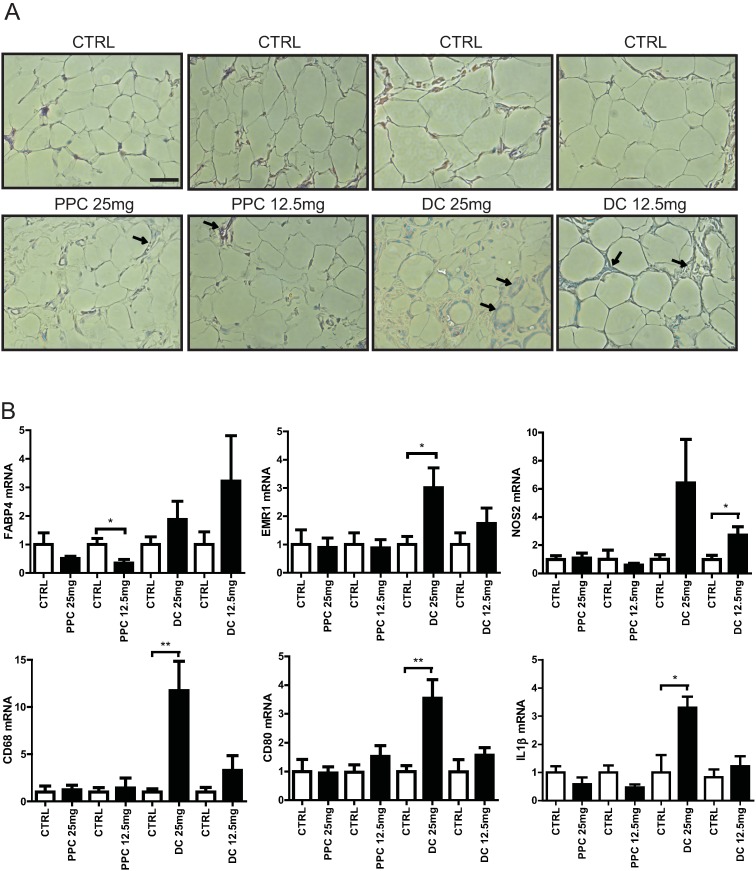
Effects of phosphatidylcholine (PPC) and deoxycholate (DC) on inguinal adipose tissue in the rat. A. Hematoxylin and eosin staining of inguinal adipose tissue sections from rats 30 days after control, PPC, or DC injection. The arrow indicates macrophage infiltration and the scale bar indicates 50 μm. B. Gene expression analysis of inguinal adipose tissue injected with control or 25 mg or 12.5 mg of PPC or DC, respectively. Using real-time qRT-PCR, the relative expression levels of FABP4, a marker for mature adipocytes and EMR1, NOS2, CD68, CD80, and IL1β markers for macrophages, in inguinal adipose tissue injected with PPC or DC were compared with the expression in inguinal adipose tissue injected with ethanol or PBS control solutions, respectively.

Rose et al. reported the recruitment of macrophages after treatment with the PPC/DC formula [15]. We investigated here which component of PPC/DC formula was responsible for the recruitment of macrophages to adipose tissue. As shown in Fig 5A, macrophage infiltration was more frequently observed in inguinal adipose tissue after both PPC and DC injection, compared to control injection. We also observed that, for both PPC and DC, injection of a higher dose induced a greater recruitment of macrophages. Interestingly, higher levels of macrophage infiltration and crown-like structures were observed in inguinal adipose tissue after DC injection, compared to PPC injection (Fig 5A and S5 Fig). Consistent with this, we also observed using qRT-PCR that the expression of macrophage markers, EMR1, NOS2, CD68, CD80, and IL1β was significantly increased in inguinal adipose tissue following DC injection (Fig 5B). We also found that mRNA expression of FABP4 was significantly decreased in samples treated with PPC. In contrast, although statistically not significant, FABP4 mRNA expression in inguinal adipose tissue showed a tendency to increase after DC treatment (Fig 5B). Together, these data imply that while DC-mediated adipose tissue reduction is non-specific, PPC contributes to the reduction of fat more specifically, inducing the death of adipocytes only.

## Discussion

Although liposuction has become a prevalent and effective procedure for the reduction of body fat, it generally requires anesthesia, and has a risk of medical complications and even death on rare occasions. Since its first application in xanthelasma treatment, injection of soybean-derived PPC has emerged as an alternative procedure for reducing localized fat deposits. In fact, several clinical trials have demonstrated that the injection of a formula comprising PPC and DC contributes to the reduction of adipose tissue volume [5–7, 16].

Previous studies have also questioned which component of the PPC/DC formula plays the bigger role in reducing fat. Rotunda et al. observed that treatments with the PPC/DC formula and with DC alone induced a comparable decrease in HaCaT cell viability and lysis of the plasma membrane. Based on this study, DC was suggested to be the principal component acting on the reduction of adipose tissue [12]. ATX-101, an injectable form of DC, was recently approved for submental fat reduction [17]. Consistent with this, our present study also showed that DC has a slightly stronger effect on the decrease of 3T3L1 preadipocyte viability (Fig 3A). However, PPC treatment appeared to have a stronger action with respect to the decrease of lipid vacuoles, which is caused by specific lysis of mature adipocytes, as demonstrated by a decrease in FABP4 protein levels after treatment with both the PPC/DC formula and purified PPC and by the live images recorded (Fig 2, S1 Fig and S3 and S4 Video files). Adipose tissue is a quite a complex and heterogeneous tissue, composed of adipocytes and stromal vascular fraction cells, which include preadipocytes, fibroblasts, vascular endothelial cells, macrophages, and mesenchymal stem cells [18]. Considering that DC is a commonly used detergent in laboratories, the reduction of adipose tissue by DC is most likely caused by the non-specific destruction of cells.

Non-surgical fat reduction procedures have many advantages as they are cost effective, require no anesthesia or hospitalization, and pose no risk of operative scars. However, safety is the most important criterion for application as a therapeutic agent. DC injections have been associated with many adverse events including pain, edema, numbness, and swelling, although these were transient and restricted to the injected sites [17]. Furthermore, patients who received DC injections complained of pain more so than those treated with the PPC/DC formula [13]. As adipose tissue is directly connected to the nervous system [19, 20], the pain after PPC/DC treatment could be caused by DC-mediated neuronal damage. Alternatively, an inflammatory response may be responsible for the pain, as increased macrophage infiltration was observed following DC treatment. The mechanism underlying this remains to be elucidated and further clinical studies, perhaps involving the scoring of the duration and intensity of pain after PPC or DC treatment, are required for verification.

The process behind the differential targeting of adipocytes by PPC is not yet clear. PPC/DC treatment has been called an injectable lipolysis procedure because it decreases fat. In physiological terms, lipolysis is the breakdown of triglycerides to glycerol and free fatty acids, and is stimulated by the adrenaline-mediated activation of ADRB3. ADRB3, by stimulating adrenylate cyclase, generates cAMP, which triggers lipolysis through PKA-dependent phosphorylation and activation of HSL [21, 22]. However, we did not see any induction of HSL-phosphorylation and compared to the ethanol treated control samples, only 20% more free fatty acid release was observed in PPC-treated samples (Fig 2 and S2 Fig). Therefore, PPC-mediated fat reduction does not seem to be mediated *via* the lipolysis pathway. Another possible mechanism could involve cell type-dependent plasma membrane proteins and phospholipid composition [23]. PPC is a major component of the plasma membrane. In adipocytes, it is the most abundant phospholipid found in the plasma membrane, especially under conditions of obesity [24]. Thus, there is a surplus of proteins that facilitate the binding or uptake of PPC, and excessive incorporation of the phospholipid into the plasma membrane of adipocytes can disintegrate the membrane structure. In line with this, we observed that adipocytes treated with PPC are apoptotic and released four-fold more triglycerides compared to adipocytes treated with the control ethanol (Fig 2F and S2 Fig). Examination of the plasma membrane phospholipid composition in 3T3L1 preadipocytes and adipocytes, and comparison of cell viability after treatment with different types of phospholipids would help to clarify this issue.

The major limitation of our study was the methodology employed to prepare the PPC solution. As PPC does not dissolve well in water, we used ethanol as the solvent. However, we only used 0.1% of ethanol (1 μl of PPC in ethanol to 1 ml of culture medium) to treat preadipocytes or adipocytes with PPC. At this concentration, ethanol itself did not affect cell viability, as samples treated with 0.1% water (the DC control) or 0.1% ethanol (the PPC control) showed a comparable cell viability. Furthermore, we compared the cell death activity of PPC with the same amount of ethanol control. Thus, the role of PPC in the induction of adipocyte cell death has been conclusively demonstrated.

Several beneficial effects of PPC have been reported, including those related to the management of hyperlipidemia, dementia, and anti-hepatic fibrosis [25–30]. Although PPC injection also appears to induce swelling, our study shows that its effects are more transient and milder than those induced by DC. Therefore, we propose PPC to be a promising agent for adipo-destruction procedures, as it acts selectively on adipocytes. Development of a better methodology for the preparation of PPC that is suitable for humans, optimization of the dose used and the interval of dosing, and combination with other treatments [31] that minimizes adverse effects should be explored for therapeutic applications.

## Supporting information

S1 Checklist (DOCX)

S1 Fig Light microscopy images of 3T3L1 preadipocytes and adipocytes eight hours after treatment with 0.025% phosphatidylcholine (PPC) or deoxycholate (DC). A. A decrease in attached 3T3L1 preadipocytes was observed, B. lipid vacuole-containing mature adipocytes were specifically decreased by PPC compared to DC treatment. The red color and blue colors indicate mature adipocytes (Oil Red O-positive) and all cells (DAPI staining), respectively. The scale bar represents 100 μm.
(PDF)

S2 Fig Light microscopy images of A. 3T3L1 preadipocytes and B. adipocytes twenty four hours after treatment with 0.025% phosphatidylcholine (PPC) or deoxycholate (DC). A. A more decrease in attached 3T3L1 preadipocytes was observed by DC treatment, B. lipid vacuole-containing mature adipocytes were specifically decreased by PPC compared to DC treatment. The scale bar represents 100 μm.
(PDF)

S3 Fig Measurement of free fatty acid and triglycerides released from differentiated adipocytes treated with PPC. A. Free fatty acid and B. triglycerides release into the cultured medium from adipocytes was measured after treatment with 0.01%, 0.05%, and 0.1% of PPC. Forskolin, which stimulates lipolysis, was used as a positive control.
(PDF)

S4 Fig Representative coronal image following MRI. Representative coronal images following MRI taken before and 7, 14, and 28 days after treatment with 25 mg phosphatidylcholine (PPC), 12.5 mg PPC, 25 mg deoxycholate (DC), or 12.5 mg DC. The left side of the white area represents control-injected and the right side of the white represents PPC- or DC-injected inguinal adipose tissue.
(PDF)

S5 Fig Increased macrophages in the inguinal adipose tissue of DC treated mice A. Immunohistochemistry showing the F4/80-positive macrophage staining of inguinal adipose tissue sections from rats 30 days after control, PPC, or DC injection. The arrow indicates macrophage infiltration (F4/80 positive staining) and the scale bar indicates 50 μm.
(PDF)

S1 Video Live confocal video files of 3T3L1 preadipocytes treated with 0.025% of phosphatidylchoine (PPC) for eight hours. (AVI)

S2 Video Live confocal video files of 3T3L1 preadipocytes treated with 0.025% of deoxycholate (DC) for eight hours. (AVI)

S3 Video Live confocal video files of differentiated adipocytes treated with 0.025% of phosphatidylchoine (PPC) for eight hours. The white dots in the cell are the lipid vacuoles of mature adipocytes.
(AVI)

S4 Video Live confocal video files of differentiated adipocytes treated with 0.025% of deoxycholate (DC) for eight hours. The white dots in the cell are the lipid vacuoles of mature adipocytes.
(AVI)
