# Supplementary figures and images for: Selective effect of phosphatidylcholine on the lysis of adipocytes
Source: PLoS One. 2017 May 2;12(5):e0176722. doi: 10.1371/journal.pone.0176722 (PMC5413042; doi:10.1371/journal.pone.0176722)

S1 Fig

A

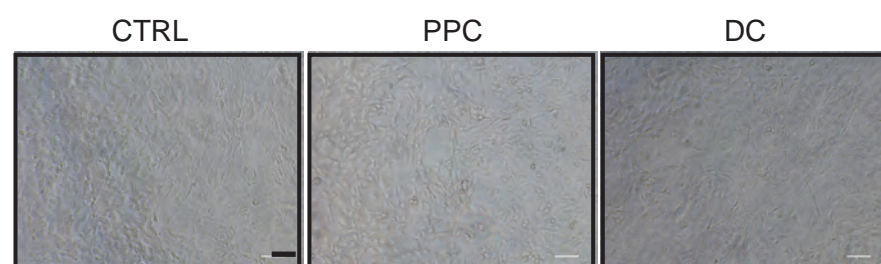

B

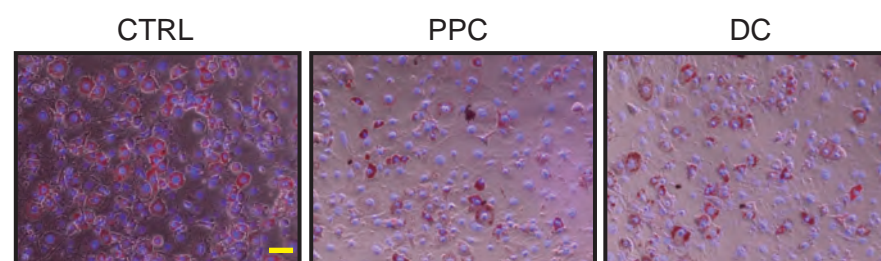

Supplement: S1 Fig — A. A decrease in attached 3T3L1 preadipocytes was observed, B. lipid vacuole-containing mature adipocytes were specifically decreased by PPC compared to DC treatment. The red color and blue colors indicate mature adipocytes (Oil Red O-positive) and all cells (DAPI staining), respectively. The scale bar represents 100 μm. (PDF) [file pone.0176722.s002.pdf]

S2 Fig

A

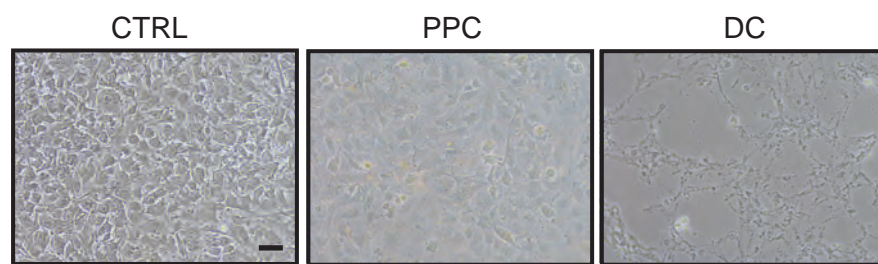

B

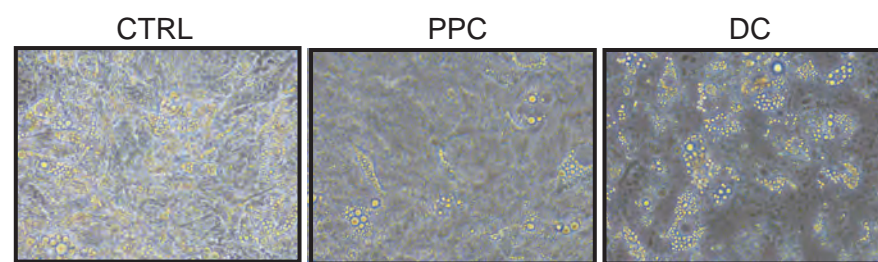

Supplement: S2 Fig — Light microscopy images of A. 3T3L1 preadipocytes and B. adipocytes twenty four hours after treatment with 0.025% phosphatidylcholine (PPC) or deoxycholate (DC). A. A more decrease in attached 3T3L1 preadipocytes was observed by DC treatment, B. lipid vacuole-containing mature adipocytes were specifically decreased by PPC compared to DC treatment. The scale bar represents 100 μm. (PDF) [file pone.0176722.s003.pdf]

S3 Fig

A

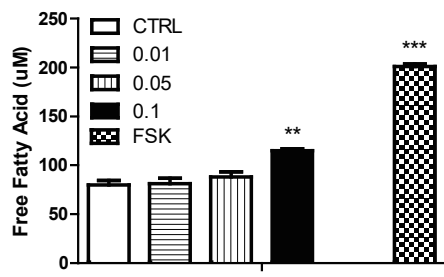

B

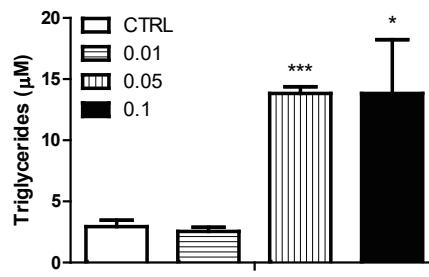

Supplement: S3 Fig — A. Free fatty acid and B. triglycerides release into the cultured medium from adipocytes was measured after treatment with 0.01%, 0.05%, and 0.1% of PPC. Forskolin, which stimulates lipolysis, was used as a positive control. (PDF) [file pone.0176722.s004.pdf]

S4 Fig

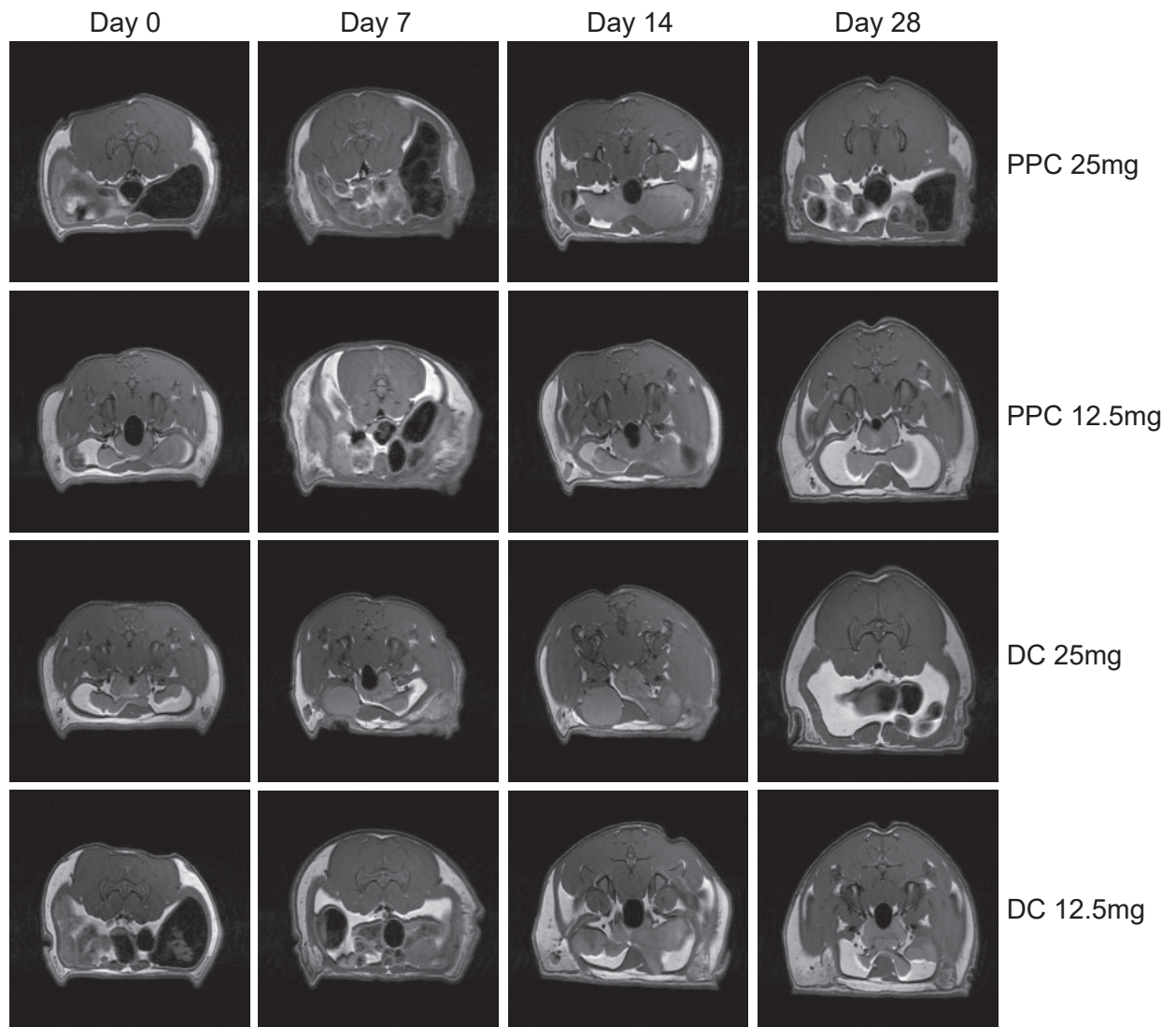

Supplement: S4 Fig — Representative coronal images following MRI taken before and 7, 14, and 28 days after treatment with 25 mg phosphatidylcholine (PPC), 12.5 mg PPC, 25 mg deoxycholate (DC), or 12.5 mg DC. The left side of the white area represents control-injected and the right side of the white represents PPC- or DC-injected inguinal adipose tissue. (PDF) [file pone.0176722.s005.pdf]

S5 Fig

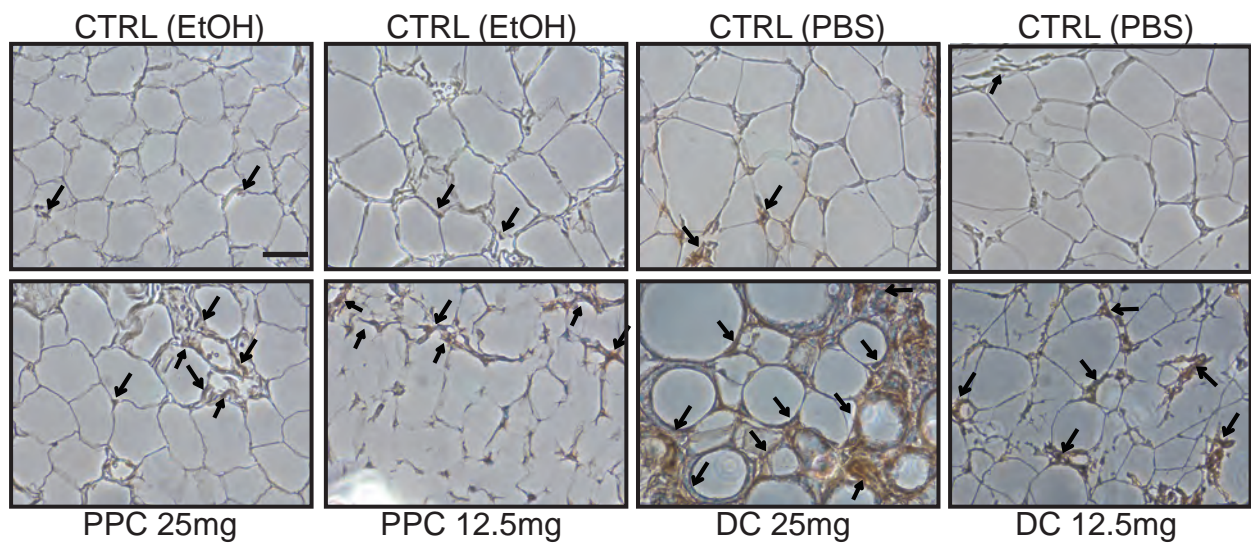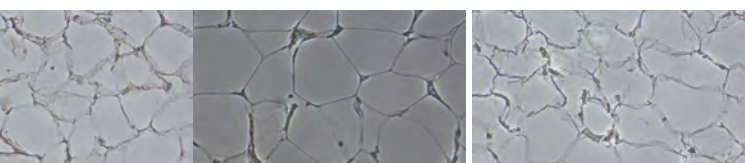

Supplement: S5 Fig — Immunohistochemistry showing the F4/80-positive macrophage staining of inguinal adipose tissue sections from rats 30 days after control, PPC, or DC injection. The arrow indicates macrophage infiltration (F4/80 positive staining) and the scale bar indicates 50 μm. (PDF) [file pone.0176722.s006.pdf]
